# Safety of Onabotulinumtoxin A in Chronic Migraine: A Systematic Review and Meta-Analysis of Randomized Clinical Trials

**DOI:** 10.3390/toxins15050332

**Published:** 2023-05-12

**Authors:** Maria Tiziana Corasaniti, Giacinto Bagetta, Pierluigi Nicotera, Assunta Tarsitano, Paolo Tonin, Giorgio Sandrini, Gary W. Lawrence, Damiana Scuteri

**Affiliations:** 1 Department of Health Sciences, University “Magna Graecia” of Catanzaro, 88100 Catanzaro, Italy; mtcorasa@unicz.it; 2 Pharmacotechnology Documentation and Transfer Unit, Preclinical and Translational Pharmacology, Department of Pharmacy, Health and Nutritional Sciences, University of Calabria, 87036 Rende, Italy; 3 German Center for Neurodegenerative Diseases (DZNE), 53127 Bonn, Germany; pierluigi.nicotera@dzne.de; 4 Pain Therapy Center, Provincial Health Authority (ASP), 87100 Cosenza, Italy; assunta.tarsitano@aspcs.it; 5 Regional Center for Serious Brain Injuries, S. Anna Institute, 88900 Crotone, Italy; patonin18@gmail.com; 6 Department of Brain and Behavioral Sciences, IRCCS C. Mondino Foundation Neurologic Institute, University of Pavia, 27100 Pavia, Italy; giorgio.sandrini@unipv.it; 7 Department of Biotechnology, Dublin City University, Collins Avenue, D09 V209 Dublin, Ireland; gary.lawrence@dcu.ie

**Keywords:** CRD42023393250, onabotulinumtoxin A, chronic migraine, safety, treatment-related adverse events (TRAEs), PRISMA 2020

## Abstract

Some 14% of global prevalence, based on high-income country populations, suffers from migraine. Chronic migraine is very disabling, being characterized by at least 15 headache days per month of which at least 8 days present the features of migraine. Onabotulinumtoxin A, targeting the machinery for exocytosis of neurotransmitters and neuropeptides, has been approved for use in chronic migraine since 2010. This systematic review and meta-analysis appraises the safety of onabotulinumtoxin A treatment for chronic migraine and the occurrence of treatment-related adverse events (TRAEs) in randomized, clinical studies in comparison with placebo or other comparators and preventative treatments according to the most updated Preferred Reporting Items for Systematic reviews and Meta-Analyses (PRISMA) 2020 recommendations. The search retrieved 888 total records. Nine studies are included and seven were eligible for meta-analysis. The present study demonstrates that toxin produces more TRAEs than placebo, but less than oral topiramate, supporting the safety of onabotulinumtoxin A, and highlights the heterogeneity of the studies present in the literature (I^2^ = 96%; *p* < 0.00001). This points to the need for further, adequately powered, randomized clinical trials assessing the safety of onabotulinumtoxin A in combination with the newest treatment options.

## 1. Introduction

Migraine is a disabling, primary headache that has a serious social impact since it represents the leading cause of years of life lived with disability in people under 50 [1]. In fact, it is estimated that some 14% globally suffers from this disease, and according to analysis of recent publications the majority come from high-income countries [2]. In agreement with the International Classification of Headache Disorders (ICHD, third revision) beta diagnostic criteria, chronic migraine is characterized by at least 15 headache days per month, of which at least 8 days present the features of migraine, for at least three months [3]. In spite of the existing treatments, lots of patients do not find adequate relief, suffering from common and long-lasting attacks of refractory migraine, impairing quality of life [4]. In fact, recent evidence highlights that some 38% of patients, having failed all available preventatives, are non-responders after 6 months of treatment to one of the newest biotechnological drugs, erenumab, a monoclonal antibody targeting the calcitonin gene-related peptide (CGRP) [5,6]. The prevalence of migraine is higher in women than in men, and for both genders it is most frequent in the 30–44 age group and lowest in the over 60 group, and around 50% of patients affected by frequent and/or severe migraine do not benefit from professional treatment [7]. Moreover, since a migraine first attack rarely occurs after the age of 50 [8,9,10], but it can turn into medication overuse headache (MOH) at over 65 [11], older patients are often excluded from clinical trials [12], particularly when subjected to the possibility of cognitive impairment [13,14,15,16] and changes in pain processing [17]. The latter issue worsened during the COVID 19 pandemic [18,19]. Onabotulinumtoxin A, targeting the machinery for the exocytosis of neurotransmitters or neuropeptides such as CGRP [20,21], has been approved for use in chronic migraine by the Food and Drug Administration (FDA) since 2010 [22], after the Phase III Research Evaluating Migraine Prophylaxis Therapy (PREEMPT) I and II (NCT00156910, NCT00168428) studies [23,24,25], and is recommended by the UK National Institute for Health and Care Excellence (NICE) for patients not responding to at least three prior preventative treatments. It provides effective, long-lasting analgesia for multiple different forms of pain, migraine and primary headache [26,27], and also reduces the need for rescue medications [28,29]. The mechanism of action of onabotulinumtoxin A consists of the inhibition of the release of neurotransmitters/neuropeptides by cleavage of the 25 kDa synaptosomal-associated protein (SNAP-25) [30]. In addition to the inhibition of neurotransmitter release from primary sensory neurons [31], mechanisms explaining its analgesic activity may include the reduction in mechanical hypersensitivity of sensitized C-units via interference with the expression of high threshold mechanosensitive ion channels [32]. In particular, there is a great deal of evidence in favor of the blockade of the neuronal release of neuromediators, such as acetylcholine in the peripheral neuromuscular junction, but also substance P and glutamate [33]. A Cochrane systematic review completed in 2018 highlighted several factors that downgrade the quality of the evidence for the efficacy of botulinum toxins in comparison with placebo or active treatment for the prevention or frequency reduction in chronic or episodic migraine: the lack of long-term data, the small size of trials with high risk of bias and unexplained heterogeneity [34]. Therefore, the aim of the present systematic review and meta-analysis is to use the updated methodology, with the incorporation of more recently available data based on all the existing randomized clinical trials up to 2 March 2023, to assess the safety of onabotulinumtoxin A in the treatment and prevention of chronic migraine. In particular, the Cochrane systematic review included randomized, controlled trials comparing botulinum toxin with placebo, active treatment or clinically relevant different doses for adults with chronic or episodic migraine, with or without the additional diagnosis of medication overuse headache, showing a relative risk of treatment-related adverse events twice that of placebo, but a reduced risk compared with active comparators. In this systematic review and meta-analysis, larger samples from the PREEMPT studies and additional studies published since 2018 are included in order to critically appraise all the existing knowledge concerned with the safety of onabotulinumtoxin A in chronic migraine following the Preferred Reporting Items for Systematic reviews and Meta-Analyses (PRISMA) 2020 guidelines.

## 2. Materials and Methods

### 2.1. Objectives, Registration and Protocol

Databases searches, screening of records and selection of results were conducted according to the most recently updated PRISMA 2020 recommendations [35,36,37]. The following participants/population, interventions, comparisons, outcomes and study design (PICOS) question has. Is there evidence for the safety of onabotulinumtoxin A in chronic migraine? Selected participants were patients suffering from chronic migraine according to the International Headache Society (IHS, version 1-2-3-3b) criteria. The intervention was onabotulinumtoxin A. The comparators examined were placebo/no treatment or active comparators. Active comparators were medications effective and approved for treatment and prevention of chronic migraine. All randomized clinical trials published prior to 2 March 2023 were eligible for inclusion in this study. Case reports/series, retrospective and non randomized clinical studies, in vivo and in vitro preclinical studies, reviews, book chapters, congress communications and proceedings were excluded. Studies not available in full text in English were excluded. Studies reporting on randomized, clinical trials assessing the safety of botulinum toxin A in chronic migraine vs. placebo or an active comparator in the title and abstract were deemed relevant for further inspection. Safety assessment was the primary outcome, measured as risk ratio (RR) to report adverse reactions. This systematic review and meta-analysis was registered, and the protocol is available on the National Institute for Health Research International prospective register of systematic reviews PROSPERO with the number CRD42023393250.

### 2.2. Information Sources

The most relevant databases for scientific and medical literature, PubMed/MEDLINE, Scopus and Web of Science (WOS), were inspected from inception to the date of last search, i.e., 2 March 2023. Duplicate records were removed using reference manager software (EndNote X7, Clarivate).

### 2.3. Search Strategy

The following MeSH terms and keywords composed search strings for the inspection of all the databases consulted to ensure high sensitivity/recall search strategy, maintaining reasonable precision without applying validated search filters: “chronic migraine”, migraine disorders”, “onabotulinumtoxin A”, “botulinum toxin A”, “botulinum toxins, type A”, “safety”, “toxicity”, “adverse reactions”, “adverse events”, “side effects” and “drug-related side effects”. The extraction of data was based on the presence of searched terms in the text, tables or graphs. In agreement with the evidence-based guideline for Peer Review of Electronic Search Strategies (PRESS) for systematic reviews [38], two review committee members independently conducted the database search (requestors) and a third independent author (reviewer) confirmed that the strategy appropriately addressed the PICOS question and the accuracy of the search strings. The requestors performed an initial pre-screen of study titles and abstracts before assessing the full texts of those that passed pre-screening for inclusion eligibility using an *a priori* set of inclusion and exclusion criteria. The reference lists of relevant papers were inspected in order to minimize the risk of missing studies eligible for inclusion. Any disagreement between the independent analysts was solved by consensus or, if necessary, by consulting a third team member.

### 2.4. Data Synthesis, Risk of Bias Assessment and Critical Appraisal

The synthesis of the results follows the Cochrane Consumers and Communication Review Group guidelines [39]. The risk of bias (RoB) and the quality/certainty [40] of the body of evidence were assessed independently by the requestors according to PRISMA 2020 statement [41]. RoB and the quality of the studies were assessed considering study limitations, a lack of allocation concealment, lack of blinding, selective outcome reporting bias, inadequate sample numbers or lack of sample size calculation. In particular, the revised Cochrane RoB2 tool for randomized, clinical trials was used [42] to reach judgements (low; some concerns; high) for each of the following specific items/domains: randomization process, deviations from intended interventions, missing outcome data, measurement of the outcome, selection of the reported result and overall RoB judgment summarizing across domains/components, considering for each study the highest level of RoB reached in each domain. The graphs of the RoB assessment were obtained using the Cochrane robvis visualization tool [43].

### 2.5. Statistical Analysis and Effect Measures

The Cochrane Review Manager 5.4.1 (RevMan5.4.1; The Nordic Cochrane Center, The Cochrane Collaboration, Copenhagen, Denmark) was used to measure risk ratio (RR) and 95% confidence intervals (CI) for dichotomous variables. The heterogeneity was measured through the random effect model [44] and the Higgins I² value [45]. The publication bias was assessed using the Egger’s linear regression test [46].

## 3. Results

### 3.1. Screening and Study Selection

**Figure 1:**
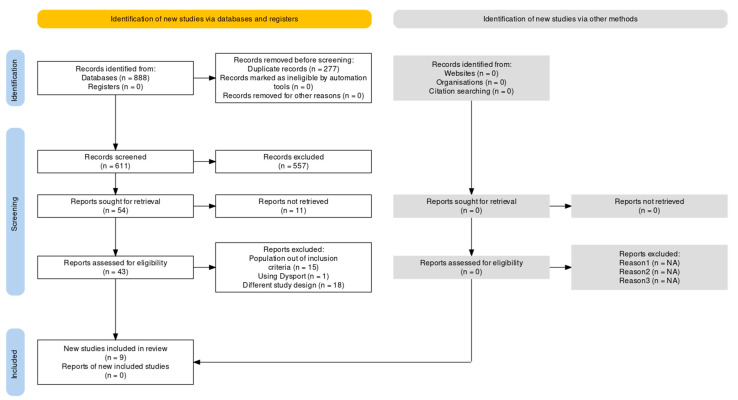
PRISMA flow diagram. Selection of records based on the Preferred Reporting Items for Systematic Reviews and Meta-Analyses (PRISMA) 2020. Flow diagram produced with the web-based Shiny app [90].

The search of PubMed/MEDLINE identified 523 records, Scopus found 128 records and WOS found 237 results. Thus, a total of 888 results were retrieved, but this was reduced to 611 records following the removal of duplicates. After title and abstract screening for inclusion criteria, 54 reports were sought for retrieval. Of these, 11 [47,48,49,50,51,52,53,54,55,56,57] were not available in full text in English, thus not meeting the eligibility criteria. After a full text screening of the remaining 43 retrieved records, 15 [51,58,59,60,61,62,63,64,65,66,67,68,69,70] were excluded because the population did not consist only of chronic migraineurs. This removed studies that included patients suffering from episodic migraine/chronic tension-type headache, migraine without specification of the frequency, or included MOH, in absence of a proper stratification of these groups. One study was excluded because it investigated the effect of a different Botulinum Type A Toxin-hemagglutinin complex preparation (Dysport) [71], whilst a further 18 studies were excluded due to incompatibility of the study design [24,72,73,74,75,76,77,78,79,80,81,82,83,84,85,86,87,88], such as the absence of quantitative reporting of safety/TRAEs, lack of randomization or the observational nature of the study. In particular, onabotulinumtoxin A contains only 900 kDa complex, whereas abobotulinumtoxin A (Dysport) is a mixture of 500 kDa and 900 kDa forms [89]. At the end of the selection process, nine studies met the inclusion criteria for the qualitative analysis and seven studies for the meta-analysis. The process of the database search and the selection of studies is illustrated in Figure 1.

### 3.2. Qualitative Synthesis

The characteristics of the nine studies eligible for qualitative analysis are detailed in Table 1. Included are studies belonging to the PREEMPT program: Aurora et al., 2011 [91] (the pooled analysis of the 56-week PREEMPT clinical program), Aurora et al., 2014 [92] (subgroup analysis of efficacy, safety and tolerability in the five treatment cycles of the PREEMPT program) and Diener et al., 2010 [25] (reporting the results of the PREEMPT 2 trial). Others fulfilling the criteria were by Cady et al., 2011 [93] (a multi-center double-blind pilot comparison of onabotulinumtoxin A and topiramate), Mathew et al., 2009 [94] (comparing onabotulinumtoxin A and Topiramate), Naderinabi et al., 2017 [95], (IRCT 201404146186N3 comparing onabotulinumtoxin A with acupuncture apart from control), Rothrock et al., 2019 [96] (FORWARD study [NCT02191579] comparing onabotulinumtoxin A and topiramate), Shehata et al., 2016 [97] (contrasting botulinum toxin with repetitive transcranial magnetic stimulation [97]) and Winner et al., 2020 [98] (investigating the use of onabotulinumtoxin A for the prevention of headaches in adolescents suffering from chronic migraine). Of the nine selected studies, only those by Naderinabi and coworkers and by Shehata and collaborators [97] were not suitable for inclusion in the meta-analysis, since they were not comparable with respect to the other seven.

### 3.3. Onabotulinumtoxin A versus Placebo

The PREEMPT clinical program compared the efficacy and safety of Onabotulinumtoxin A (155–195 U) with a placebo. In the study by Aurora et al., 2011 [91], 4.8% of patients in the onabotulinumtoxin A group experienced a serious adverse event during the double-blind phase compared to 2.3% for the placebo group. The incidence for individual TRAEs was consistent with the established safety of onabotulinumtoxin A after injection into head and neck muscles. Neck pain in the onabotulinumtoxin A group occurred in 6.7% of patients. Total TRAEs occurred in *n* = 202 (29.4%) patients in the onabotulinumtoxin A group and in *n* = 88 (12.7%) patients in the placebo group. Discontinuation before week 56 due to AEs occurred in *n* = 38 (5.5%) patients in the onabotulinumtoxin A group and in *n* = 26 (3.7%) patients in the placebo group. In the later study by Aurora et al., 2014 [92], TRAEs were experienced by *n* = 147 (28.5%) of the onabotulinumtoxin A group and by *n* = 61 (12.4%) of the placebo group in the double-blind phase. The most frequently reported TRAEs in patients receiving all the five treatment cycles of onabotulinumtoxin A were reported to be neck pain (4.3%), muscular weakness (1.6%), injection site pain (2.1%) and eyelid ptosis (1.9%). Furthermore, the rate of TRAEs progressively decreased over the treatment with onabotulinumtoxin A, strengthening the evidence for the safety and tolerability of this treatment. The study by Diener and collaborators, 2010 [25], highlighted TRAEs in *n* = 116 (33.4%) of onabotulinumtoxin A-receiving patients and in *n* = 49 (13.7%) from the placebo-receiving patients. The severity of most AEs experienced was mild or moderate and reversible without sequelae. The only TRAEs in the onabotulinumtoxin A group with a rate of presentation over 5% were neck pain (7.5%) and muscular weakness (5.2%). Other TRAEs included eyelid ptosis, myalgia and musculoskeletal stiffness, without the occurrence of any newly emerged safety findings. The only serious TRAE reported for onabotulinumtoxin A was migraine requiring hospitalization. In the study by Winner et al., 2020 [98], no patient was reported to have discontinued because of AEs. TRAEs occurred in ten (23%) patients of the onabotulinumtoxin A 155 U group, in seven (16%) patients of the onabotulinumtoxin A 74 U group and in four (11%) patients from the placebo group. The severity of most TRAEs was reported to be mild in the onabotulinumtoxin A 155 U (eight patients = 80%) and onabotulinumtoxin A 74 U (five patients = 71%) groups, as in the placebo group (two patients = 50%). TRAEs with rate over 3% in onabotulinumtoxin A group were neck pain (five patients = 6%) and musculoskeletal pain (four patients = 5%) without evidence of a dose–response relationship. None of the TRAEs that occurred were associated with the possible distant spread of onabotulinumtoxin A, in spite of one case of facial paresis due to a local effect. This demonstrates the safety and local action of onabotulinumtoxin A in chronic migraine.

### 3.4. Onabotulinumtoxin A versus Topiramate

In the study by Cady et al., [93] comparing onabotulinumtoxin A injections plus placebo tablets with topiramate plus placebo injections, the AEs experienced by the intervention group consisted of mild fatigue, nausea, difficulty concentrating, memory loss and mood swings. In particular, a difference between groups was reported for nausea, occurring in 59.1% patients treated with onabotulinumtoxin A in comparison with 27.3% patients receiving topiramate. Discontinuation occurred for fifteen patients, eight of whom belonged to the topiramate group and seven to the onabotulinumtoxin A group, with only half of these patients dropping out because of AEs. In the study performed by Mathew and colleagues, 2009 [94], the safety of onabotulinumtoxin A was confirmed since TRAEs occurred in 18 (69.2%) patients from the onabotulinumtoxin A group and in 25 (86.2%) patients from the topiramate group. In particular, safety and tolerability were evaluated assessing the frequency and severity of AEs and premature withdrawal from the study, demonstrating the following findings: most AEs were mild to moderate; the study was completed at month 9 by 63.3% (19/30) of patients in the onabotulinumtoxin A group and by 56.7% (17/30) of patients in the topiramate group, with only three and eight patients in the onabotulinumtoxin A and topiramate groups, respectively, discontinuing because of AEs. In the FORWARD study by Rothrock et al., 2019 [96], TRAEs occurred in 17% of patients treated with onabotulinumtoxin A, 70% of patients treated with topiramate, and 15% of patients that voluntarily crossed over to onabotulinumtoxin A after 12 weeks’ treatment with topiramate. The most common TRAEs in the group receiving onabotulinumtoxin A were neck pain (4%), musculoskeletal pain (2%), migraine (1%) and blurred vision (1%). By contrast, the most common TRAEs in patients receiving topiramate were paresthesia (29%), cognitive disorder, fatigue, nausea (all 12%), decreased appetite, dizziness (both 11%) and attention disturbance (8%). The only serious TRAE observed was nephrolithiasis in a patient treated with topiramate, and there were no cases of death.

### 3.5. Onabotulinumtoxin A versus Non-Pharmacological Comparators

Naderinabi and collaborators, 2017 [95], compared three treatments: onabotulinumtoxin A, acupuncture and control (sodium valproate 500 mg/day). Incidence of nausea and vomiting did not differ among the three groups within two months of follow-up (*p* > 0.05), but after three months the botulinum toxin type A group presented a higher incidence (*p* = 0.027). A significantly lower rate of AEs was recorded for the acupuncture group compared to the group receiving botulinum toxin (6% vs. 22%; *p* = 0.021). The TRAEs of acupuncture consisted of bleeding or subcutaneous hematoma formation. The TRAEs of botulinum toxin A included ptosis, facial masking or asymmetry. The TRAEs reported in the sodium valproate group after 3 months included asthenia in five (10%) patients, anorexia in two (4%), weight gain in two (4%), tremor in nine (18%), somnolence in nine (18%), insomnia in four (8%) and alopecia in seven (14%). The study by Shehata et al., 2016, compared onabotulinumtoxin A with repetitive transcranial magnetic stimulation (rTMS) without reporting any systemic reactions or serious AEs.

The frequency of AEs relative to the comparators is reported in Table 2.

### 3.6. Critical Appraisal

**Figure 2:**
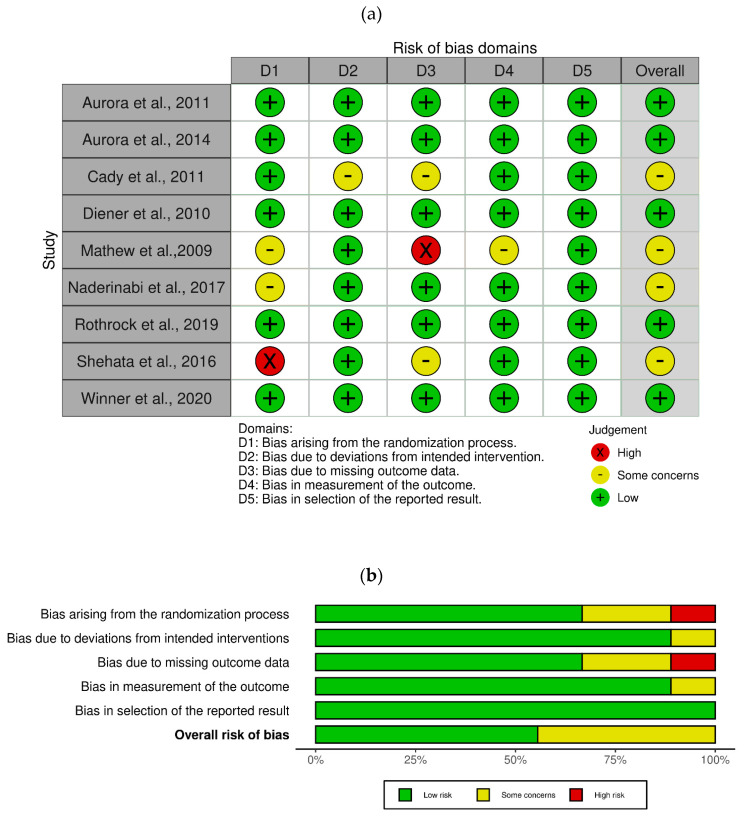
Study assessment according to the revised Cochrane RoB2 tool for randomized clinical trials. The Cochrane robvis visualization tool [43] was used to present RoB as (**a**) a traffic-light plot and (**b**) weighted bar plots.

The RoB assessment evaluated the following five domains: (1) the randomization process; (2) deviations from the intended interventions, considering the effect of assignment to intervention and the effect of adhering to intervention; (3) missing outcome data, assessing attrition bias and loss at follow-up; (4) measurement of the outcome; and (5) selection of the reported results. In the study of Aurora et al., 2011 [91], no bias for randomization or allocation occurred and only three cases of protocol violation were detected; therefore, no bias existed in terms of deviations from the intended protocol. However, baseline differences in the mean frequency of total cumulative hours of headache occurring on headache days and in the mean frequency of headache and migraine episodes were identified. Since discontinuation occurred in *n* = 38 (5.5%) patients in the onabotulinumtoxin A group and in *n* = 26 (3.7%) patients in the placebo group, almost all the patients out of the total presented the outcome measures; thus, there was no missing data bias. In the study by Aurora et al., 2014 [92], there was no concern in terms of RoB, or for baseline since patients were stratified by whether or not they overused acute headache medication during the 28-day baseline and because there were no statistically significant differences in baseline characteristics apart from the mean frequency of headache days and for cumulative hours of headache occurring on headache days. Although the average baseline headache characteristics were similar, the study by Cady et al., 2011., [93] presented some concerns of RoB in terms of domains 2 (deviations from intended interventions) and 3 (missing data). In fact, the sample was small and there was a high number of dropouts in comparison with the sample size. In the study by Diener and coworkers, 2010 [25], all randomized patients receiving at least one dose of study medication at day 0 were subjected to safety analyses. Moreover, patients were randomized 1:1 in a double-blind manner to onabotulinumtoxin A or placebo and stratified according to the frequency of acute headache pain medication use and overuse at baseline in blocks of four within each medication–overuse stratum for each investigator site. There were no statistically significant baseline characteristics apart from mean headache episodes during the 28-day baseline, and only one (0.3%) deviation from the intended protocol was reported in the onabotulinumtoxin A group. Therefore, there was no concern in terms of RoB. In the study conducted by Naderinabi and coworkers, 2017 [95] (IRCT 201404146186N3), patients were randomly allocated to groups through designed quadripartite blocks and no statistically significant differences in baseline were reported. The physician rating the data was blinded to the type of treatment. In the study by Mathew et al., 2009 [94], patients in the onabotulinumtoxin A and topiramate groups differed only for age at onset of migraine (14.9 ± 7.2 and 20.0 ± 9.2 years for the onabotulinumtoxin A and topiramate groups, respectively, with *p* = 0.0151). It was reported that three patients performed the final month 9 visit, albeit earlier than scheduled; therefore, no significant deviations from the intended interventions occurred. However, the high dropout rate caused a high RoB in domain 3 (missing data). In the study by Rothrock et al., 2019 [96], 11 (8%) patients initially randomized to the onabotulinumtoxin A group and 89 (63%) patients randomized to topiramate discontinued the trial. Missing values were imputed using baseline observation carried forward (BOCF) methodology, considered at low RoB [34]. The study by Shehata [97] was open-label, giving rise to a high RoB in domain 1 (randomization), and dropout rates raised some concerns in domain 3 (missing data). The study by Winner et al., 2020 [98] was randomized, double-blind and had sample size calculation. Treatment groups were well-balanced in terms of baseline characteristics, and only one protocol violation in the 155U group was reported. No patient was reported to have discontinued because of AEs. Hence, no RoB occurred. Considering adverse events reporting, no outcome measure bias or selective reporting were detected in any of the trials. The RoB assessment as a traffic-light (Figure 2a) and weighted bar (Figure 2b) plots is reported in Figure 2.

### 3.7. Meta-Analysis

**Figure 3:**
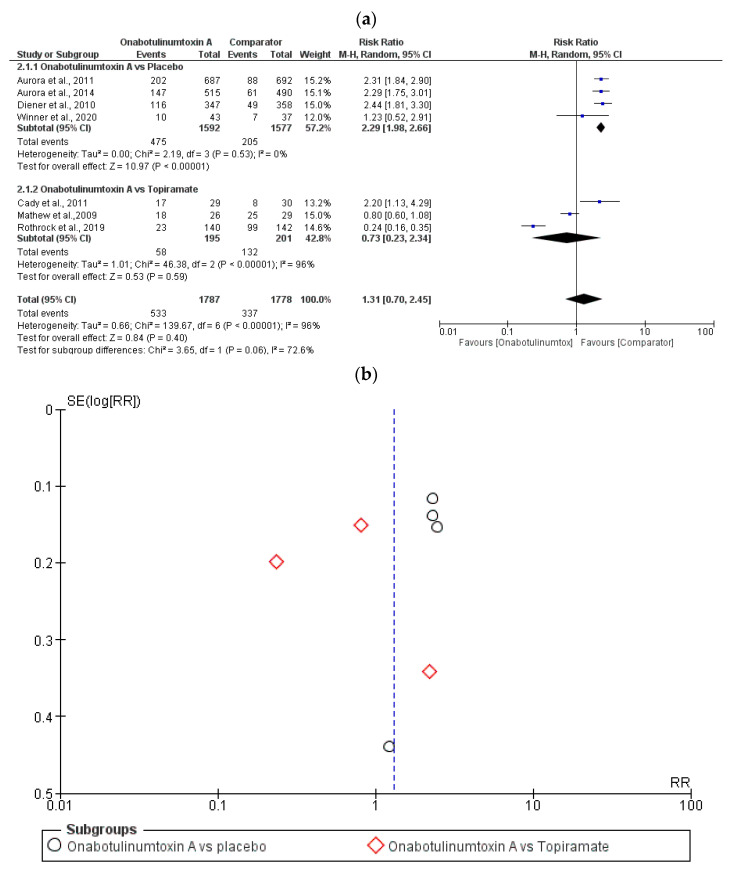
Toxin produces more treatment-related adverse events (TRAEs) than placebo, but less than oral topiramate and the presence of publication bias: forest plot of the risk ratio (RR) of the studies eligible for meta-analysis for a sample of *n* = 1787 patients treated with onabotulinumtoxin A and *n* = 1778 patients treated with a comparator (placebo or topiramate) (**a**); (**b**) funnel plot for publication bias [25,91,92,93,94,96,98].

Meta-analysis was performed on *n* = 1787 patients treated with onabotulinumtoxin A and *n* = 1778 patients treated with a comparator (also including pooled and subgroup analyses from PREEMPT program). A forest plot (Figure 3a) with subgroup analysis for comparator, i.e., placebo or topiramate, demonstrates that toxin produced more TRAEs than placebo, but fewer than oral topiramate. The studies by Mathew et al., 2009 [94], and by Winner et al., 2020 [98], crossed the line of null effect, influencing the overall result, because of their weight in the analysis due to the sample size. In agreement with the diamond placement, the total result was not statistically significant for the outcome of interest of the meta-analysis (*p* = 0.40), as supported by the high heterogeneity of the studies (I^2^ = 96%; *p* < 0.00001), due to study sample size and to variations in the comparators, mainly. Therefore, the overall result of the forest plot is questionable, and overestimated for the effect of the intervention on the outcome of the meta-analysis. Moreover, there is evidence of publication bias supported by the funnel plot (Figure 3b), with an asymmetrical appearance and a gap in the right bottom side of the graph demonstrating that smaller studies are missing [99].

## 4. Discussion

Migraine is the second largest contributor to disability-adjusted life-years (DALYs) according to the estimates of the Global Burden of Disease Study 2016 [100]. The clinical relevance of difficult-to-treat migraine is highlighted by the high proportion of physicians reporting frequent visits to their practice from resistant and refractory migraineurs [6]. Onabotulinumtoxin A has been approved since 2010 for the treatment of chronic migraine. The present systematic review and meta-analysis shows the safety of onabotulinumtoxin A treatment in comparison with an oral preventative of common use, topiramate, but it highlights the paucity of randomized, clinical trials in the field. Indeed, application of the PRISMA 2020 workflow selected only nine studies [25,91,92,93,94,95,96,97,98] in the qualitative synthesis and seven in the meta-analysis out of the 888 studies identified through a search of the PubMed/MEDLINE, Scopus and WOS databases. It was noticeable that there was an occurrence of overall consensus among the authors, without relevant conflicts planned to be solved through the Delphi method [101]. The retrieved records proved that only a few randomized, placebo-controlled and double-blind clinical trials assessing the safety and tolerability of onabotulinumtoxin A exist, indicating that the interest in the rigorous study of the efficacy and safety of this therapy for chronic migraine declined after its approval. Moreover, studies performed after the PREEMPT clinical program used much lower sample numbers. For this reason, a limitation of this study is the inclusion of data from the PREEMPT clinical program, as well as its pooled and subgroup analysis, in the quantitative synthesis [25,91,92]. Although all the included trials shared similar study designs, they compared the intervention to multiple different comparators (placebo, topiramate or non-pharmacological approaches), introducing variability into the measurements. Furthermore, total heterogeneity (I^2^ = 96%; *p* < 0.00001) originating from differences in the size of the study samples was compounded by high rates of dropout. The trials with the smallest sample size or heterogeneity due to comparator and design characteristics influencing the overall result were the studies conducted by Mathew et al., 2009 [94], Naderinabi et al., 2017 [95], and Shehata et al., 2016 [97]. The main causes of RoB consisted of bias arising from the baseline data, the sample size, the deviations from intended protocol and the missing outcome data. In the reported sample including *n* = 1787 patients treated with onabotulinumtoxin A and *n* = 1778 patients treated with a comparator, TRAEs associated with the intervention using onabotulinumtoxin A were mild to moderate and resolved without sequelae, most often including neck pain, musculoskeletal pain, muscular weakness, migraine, eyelid ptosis, blurred vision and injection site pain. Therefore, the results of the present analysis confirm that the occurrence of TRAEs associated with onabotulinumtoxin A are consistent with its established safety after injection into head and neck muscles. In fact, it is fundamental to underline the mild nature of the TRAEs induced by the treatment with onabotulinumtoxin A and their fast and spontaneous resolution without complications. In most cases, botulinum toxin is well-tolerated and the side effects are rare, mild and self-limiting in a short time. Combination therapy, utilizing injections of both onabotulinumtoxin A and antibodies targeting CGRP or its receptor, is emerging as a potential new treatment paradigm for intractable chronic migraine. In fact, a pooled analysis from our group [102] highlights the lack of use of onabotulinumtoxin A and that the combined treatment affords ≥50% reduction of monthly headache days/frequency with respect to baseline in up to 58.8% of patients, with a pooled percentage decline of 35.5% after 6 months. Strikingly, these results show a better efficacy than the monoclonal antibody erenumab, alone or in combination with other prophylactic treatments, proving efficacy in only 26% and 15% of patients, respectively. Moreover, this combination has the potential to extend the therapeutic benefit and to delay the wear-off by an average of two weeks [102] to meet the needs of resistant patients [103]. Future, homogeneous studies are needed to affirm the efficacy and safety of onabotulinumtoxin A administered in combination with monoclonal antibodies targeting CGRP signaling compared to either treatment alone or to placebo. The possible synergistic/additive effect of the combination of onabotulinumtoxin A and anti-CGRP monoclonal antibodies can involve not only the CGRP machinery [104], but all the following mechanisms: a reversal of mechanical hypersensitivity of sensitized C-units; the neuronal/Schwann cell pathway [105]; and the blocking of the release of several neurotransmitters, e.g., acetylcholine, glutamate and substance P, by botulinum neurotoxins. Monoclonal antibodies can act on Aδ- but not C-fibers, in contrast with the toxin that acts on C- but not Aδ-fibers [106]. Critical neuromediators in this process are substance P, glutamate, fractalkine and inflammatory cytokines, the restoration of the neuroimmune balance in favor of the reduction of NF-κB, p38 and ERK1/2 phosphorylation in microglia and of interaction with Toll-like receptor 2 (TLR2) and its adaptor protein MyD88 [107,108,109,110]. Another important aspect to consider is the narrow population recruited for clinical trials in migraine, often excluding patients over 65, as for other drugs [12], to obtain pharmacovigilance information about polypharmacy [111,112] and any impact of drug-to-drug [113]/herbal medicines interactions [114] and with nutraceuticals [115]. In fact, phytocomplexes endowed with analgesic and non-benzodiazepine-like anxiolytic effects [116,117,118,119,120,121,122,123,124] deserve investigation for pain [125] as an add-on in these patients, to reduce AEs particularly in the light of the neuropsychiatric symptoms developed [126,127,128]. Furthermore, data concerned with the safety of onabotulinumtoxin A with concomitant antithrombotic therapy in patients with muscle spasticity have been provided in a retrospective, pooled analysis of randomized sponsored studies investigating onabotulinumtoxin A for the treatment of post-stroke upper or lower limb muscle spasticity [129]. The results demonstrate that the incidence of bleeding was 0.9% in patients receiving antithrombotic therapy after the treatment with onabotulinumtoxin A (with respect to 1.4% in patients not receiving antithrombotic therapy). Therefore, these data also support the safety of onabotulinumtoxin A in a course of antithrombotic therapy.

## 5. Conclusions

The present study sheds light on the need for adequately powered, randomized, clinical trials with a wider study sample assessing the efficacy and safety of onabotulinumtoxin A, and also in combination with anti-CGRP monoclonal antibodies and the newest CGRP receptor antagonists [130,131] in chronic migraine, in the view of the small sample size, often-retrospective design and heterogeneity in terms of the outcomes chosen in the actually existing literature [132].

## Figures and Tables

**Table 1:** Summary of the characteristics and of the findings for safety outcome of the studies included in the qualitative analysis.

Study	Aurora et al., 2011 [91]	Aurora et al., 2014 [92]	Cady et al., 2011 [93]	Diener et al., 2010 [25]	Mathew et al., 2009 [94]	Naderinabi et al., 2017 [95]	Rothrock et al., 2019 [96]	Shehata et al., 2016 [97]	Winner et al., 2020 [98]
**Study design**	Phase III REsearch Evaluating Migraine Prophylaxis Therapy (PREEMPT) clinical program—Pooled analysis	Phase III REsearch Evaluating Migraine Prophylaxis Therapy (PREEMPT) clinical program—Subgroup analysis	Multi-center double-blind pilot trial	Phase III REsearch Evaluating Migraine Prophylaxis Therapy (PREEMPT) 2 clinical trial	Single-center, double-blind trial	Iranian Registry of Clinical Trial IRCT 201404146186N3 randomized clinical trial	Multicenter, randomized, parallel-group, post-authorization, open-label prospective study (FORWARD; Clinicaltrials.gov NCT02191579)	Pilot, randomized trial on a small-scale	Multicenter, double-blind, parallel-group, randomized trial
**Intervention**	Onabotulinumtoxin A (155–195 U)-pooled analysis of two phase III, 24-week, double-blind, parallel-group, placebo-controlled studies, followed by a 32-week, open-label, single-treatment, onabotulinumtoxin A phase. *n* = 687	Onabotulinumtoxin A (155–195 U)-secondary analysis assessed patients who received all five treatment cycles. *n* = 515	Onabotulinumtoxin A injections plus placebo tablets. Up to 200 units of onabotulinumtoxin A or placebo injected with 100 units into fixed locations and up to additional 100 units in a follow-the-pain scheme determined by the investigator. Average dosage of 109 units for the first injection cycle. *n* = 29	Onabotulinumtoxin A (155–195 U)-Phase 3 study, with a 24-week, double-blind, placebo-controlled phase, followed by a 32-week, open-label phase. *n* = 347	Onabotulinumtoxin A, maximum 200 units (U) and 100 U fixed-site and 100 U follow-the-pain at month 3. *n* = 26	Onabotulinumtoxin A 155U following the protocol of the Phase ΙΙΙ Research Evaluating Migraine Prophylaxis Therapy I (PREEMPT1). *n* = 50	Onabotulinumtoxin A 155 U every 12 weeks for 3 cycles. *n* = 140	Onabotulinumtoxin A following the Phase III Research Evaluating Migraine Prophylaxis Therapy injection paradigm. *n* = 15	Single treatment of onabotulinumtoxin A (155 U or 74 U) fixed-dose and fixed-site paradigm. *n* = 43
**Comparator**	Placebo *n* = 692	Placebo *n* = 490	Topiramate plus placebo injections. Initiated at 25 mg daily and escalated to 100 mg in weekly incremental changes of 25 mg. The dosage can be further escalated after one month to a maximum dosage of 200 mg per day. Average daily dosage of 136 mg by week 12. *n* = 30	Placebo *n* = 358	Topiramate (4-week titration to 100 mg/day with option for additional 4-week titration to 200 mg/day, plus placebo injections. *n* = 29	Acupuncture group (*n* = 50) and control group (sodium valproate 500 mg/day; *n* = 50)	Topiramate “immediate release” 50–100 mg/day to week 36 (*n* = 142). After 12 weeks, patients initially randomized to topiramate can cross over to onabotulinumtoxin A treatment (*n* = 80)	Repetitive transcranial magnetic stimulation (rTMS). *n* = 14	Placebo. *n* = 37
**Summary of safety findings**	The proportion of patients experiencing a serious adverse event during the double-blind phase was 4.8% for onabotuli-numtoxin A group and 2.3% for the placebo group. The incidence of individual treatment-related adverse events (TRAEs) was 6.7% in the onabotulinumtoxin A group. Total TRAEs occur in *n* = 202 (29.4%) patients in the onabotulinumtoxin A group and in *n* = 88 (12.7%) patients in the placebo group	TRAEs were experienced by *n* = 147 (28.5%) of onabotulinumtoxin A group and by *n* = 61 (12.4%) of placebo group in the double-blind phase	Nausea occurred in 59.1% patients treated with onabotulinumtoxin A in comparison with 27.3% patients receiving topiramate	TRAEs in *n* = 116 (33.4%) of onabotulinumtoxin A-receiving patients and in *n* = 49 (13.7%) from the placebo-receiving patients	TRAEs occurred in 18 (69.2%) patients from the onabotulinumtoxin A group and in 25 (86.2%) patients from the topiramate group	Significant lower rate of AEs was recorded for the acupuncture group rather than the group receiving botulinum toxin (6% vs. 22%; *p* = 0.021)	TRAEs occurred in 17% of patients treated with onabotulinumtoxin A and in 70% of patients treated with topiramate	No systemic reactions or serious AEs were recorded	TRAEs occurred in 10 (23%) patients in the onabotulinumtoxin A 155 U group, in 7 (16%) patients in the onabotulinumtoxin A 74 U group and in 4 (11%) patients in the placebo group

**Table 2:** Frequency of adverse events induced by onabotulinumtoxin A relative to placebo, topiramate and other comparators.

Onabotulinumtoxin A vs.	Placebo	Topiramate	Other Comparators
**Frequency of adverse events** (% of patients experiencing the adverse event in the onabotulinumtoxin A group)	Neck pain (4.3–7.5%), muscular weakness (1.6–5.2%), musculoskeletal pain (5%), injection site pain (2.1%) and eyelid ptosis (1.9%).	Nausea (59.1%), neck pain (4%), musculoskeletal pain (2%), migraine (1%) and blurred vision (1%).	Ptosis, facial masking or asymmetry (22%; respect to acupuncture and sodium valproate); No serious adverse event with respect to repetitive transcranial magnetic stimulation.

## Data Availability

Datasets analyzed or generated during the study are available in the text.
